# Singlet fission in a hexacene dimer: energetics dictate dynamics†

**DOI:** 10.1039/c9sc05066c

**Published:** 2019-12-09

**Authors:** Samuel N. Sanders, Elango Kumarasamy, Kealan J. Fallon, Matthew Y. Sfeir, Luis M. Campos

**Affiliations:** Department of Chemistry, Columbia University New York NY 10027 USA lc2730@columbia.edu; Photonics Initiative, Advanced Science Research Center, City University of New York New York NY 10031 USA msfeir@gc.cuny.edu; Department of Physics, Graduate Center, City University of New York New York NY 10016 USA

## Abstract

Singlet fission (SF) is an exciton multiplication process with the potential to raise the efficiency limit of single junction solar cells from 33% to up to 45%. Most chromophores generally undergo SF as solid-state crystals. However, when such molecules are covalently coupled, the dimers can be used as model systems to study fundamental photophysical dynamics where a singlet exciton splits into two triplet excitons within individual molecules. Here we report the synthesis and photophysical characterization of singlet fission of a hexacene dimer. Comparing the hexacene dimer to analogous tetracene and pentacene dimers reveals that excess exoergicity slows down singlet fission, similar to what is observed in molecular crystals. Conversely, the lower triplet energy of hexacene results in an increase in the rate of triplet pair recombination, following the energy gap law for radiationless transitions. These results point to design rules for singlet fission chromophores: the energy gap between singlet and triplet pair should be minimal, and the gap between triplet pair and ground state should be large.

## Abstract 2

We report the synthesis and photophysical characterization of highly exoergic singlet fission in a hexacene dimer revealing exciton dynamics that follow the energy gap law.

## Graphical abstract

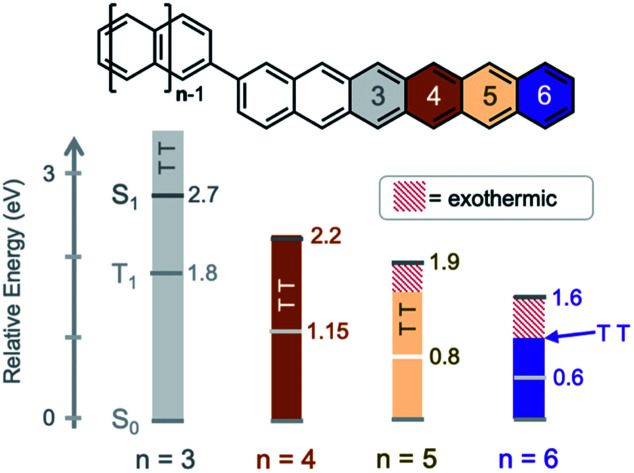

## Introduction

The potential to exploit exciton multiplication in a variety of applications has sparked interest to develop materials to understand intrinsic fundamental details of excited state dynamics.^1–7^ Singlet fission, where one photon produces two excitons, can occur in organic chromophores with energetically low-lying triplet states.^8,9^ This process requires electronic interaction between two or more chromophores, and so most research has focused on molecular crystals, polymers, or dimer assemblies in solution.^10–18^ Dimers serve as model systems to study singlet fission. They represent the fundamental smallest number of chromophores required for SF and varying the connectivity between the chromophores can lead to insightful structure–property relationships of the constrained excitons, from the generation,^19–22^ separation,^23–26^ and recombination^27–30^ of triplet states, to the elucidation of the bound triplet pair state.^31–34^

It has been established that molecular vibrations play a key role in mediating singlet fission in both oligoacene molecular crystals (intermolecular singlet fission, xSF) and in acene intramolecular singlet fission (iSF) compounds.^17,35–38^ In crystals, it is now commonly accepted that both coherent and incoherent formation of triplet pairs is possible, even within the same system.^35,39^ Experimental signatures of vibrational coherences have been detected using ultrafast vibrational and 2D electronic spectroscopy, where both inter- and intramolecular vibrational modes have been found to be important.^40–44^ For incoherent triplet pair formation, the signature of vibrational mediation has been the dependence of the singlet fission rate constant on the energetic driving force Δ*E*_S-TT_. This driving force increases with *n*, the number of rings in the oligoacene chromophore, such that tetracene (Tc, *n* = 4) < pentacene (Pc, *n* = 5) < hexacene (Hc, *n* = 6). However, the singlet fission rate constant is not monotonic with Δ*E*_S-TT_, being maximized when the driving force approaches zero and decreasing for more exo- or endothermic conditions. The rate constant decreases considerably in hexacene, where Δ*E*_S-TT_ is on the order of several molecular vibrations.^36,45,46^

The important role of vibrations in iSF has also been explored.^17^ However, no coherent generation of triplet pairs has been reported to date. Rather, singlet fission has been shown to be a purely incoherent process, which can span any time scale that can kinetically compete with decay of the photoexcited singlet. In bridged molecular dimers, singlet fission time constants on the order of 10 ns have been reported.^47,48^ Recent calculations have suggested that molecular vibrations are essential to bring the energy of the singlet and triplet pair into resonance, enabling fast SF.^17^ As such, we would expect a similar dependence of the singlet fission rate constant as a function of driving force, *i.e.*, as the energy difference between the singlet and triplet pair increases, the probability of overcoming this energy difference by coupling to molecular vibrations should decrease. However, no iSF materials with a large energetic driving force have been reported to date that would allow us to test this hypothesis.

While dimers of tetracene and pentacene have been extensively studied, the excited state dynamics of hexacene dimers (and longer oligoacenes, *n* > 6) remain unknown. This is not surprising given that the stability of the oligoacenes is compromised as their π-system is extended and it has been a major challenge to stabilize heptacene (*n* = 7)^49^ as calculations predict the emergence of an open-shell diradical character of the singlet ground-state.^50,51^

Additionally, oligoacenes with low triplet energies are needed to understand triplet pair decay processes. A large range of triplet pair lifetimes has been observed in iSF compounds. Coupled triplet pairs in contiguous molecular dimers have been shown to undergo a rapid non-radiative geminate decay process, while coupled triplet pairs in bridged iSF compounds have been found to persist for ∼1 μs.^52^ While recently reported molecular design schemes have shown ways to suppress fast recombination and permit quantitative generation of free triplets in individual molecules,^23^ the nature of this phenomena is still unexplained. Preliminary data has showed that concerted triplet pair decay follows the energy gap law for radiationless transitions, suggesting that multi-vibration relaxation to the singlet ground state is directly occurring.^29^ Nonetheless, a wider range of triplet pair energies are needed to establish this fact. Thus, in order to develop general guidelines for the design and synthesis of SF chromophores, it is imperative to understand how the intrinsic energies of the materials impact the formation, dissociation, and recombination of multiple exciton states in individual molecules.

**Fig. 1:**
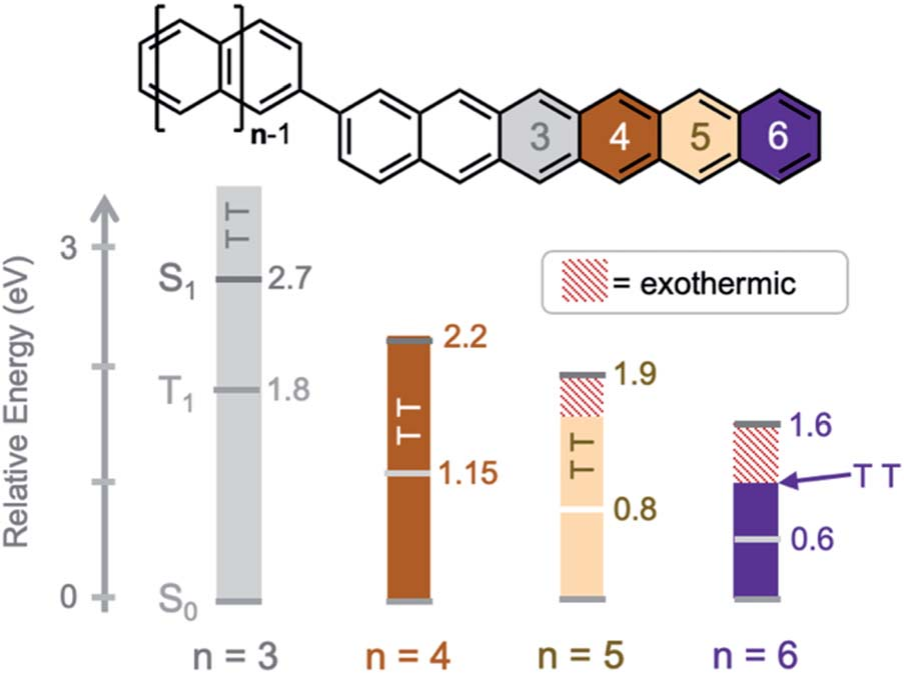
Increasing the exothermicity of singlet fission by extension of the fused acene units. Singlet fission is isoergic in tetracene (*n* = 4), exothermic in pentacene, and highly exothermic in hexacene. The *T*_1_ and S_1_ energies are marked by light/dark gray lines and the triple pair (TT) energy is given by solid bars (solubilizing/stabilizing groups omitted for clarity).

Here, we study the excited state dynamics of a hexacene dimer (bihexacene, **BH**, *n* = 6) – the most exothermic known SF chromophore of the oligoacene series. Moreover, we compare the excited state dynamics of bitetracene (**BT**, *n* = 4), bipentacene (**BP**, *n* = 5), and pentacene–tetracene and pentacene–hexacene heterodimers (**PT**, **PH**) that have similar core connectivity and stabilizing/solubilizing groups. Within this series, both the energetic driving force for singlet fission and the total triplet energy changes significantly (Fig. 1) using triplet energies taken from literature (summary in ESI†).^53,54^ These studies provide fundamental insights into the role of vibrations in mediating both the formation and decay of triplet pairs. We find that an energy gap law^55,56^ holds for both the formation and decay of triplet pairs, with the magnitude of the rate constants decreasing when an increasing number of molecular vibrations is required to overcome energetic differences between the singlet and triplet pair potential energy surfaces.

**Fig. 2:**
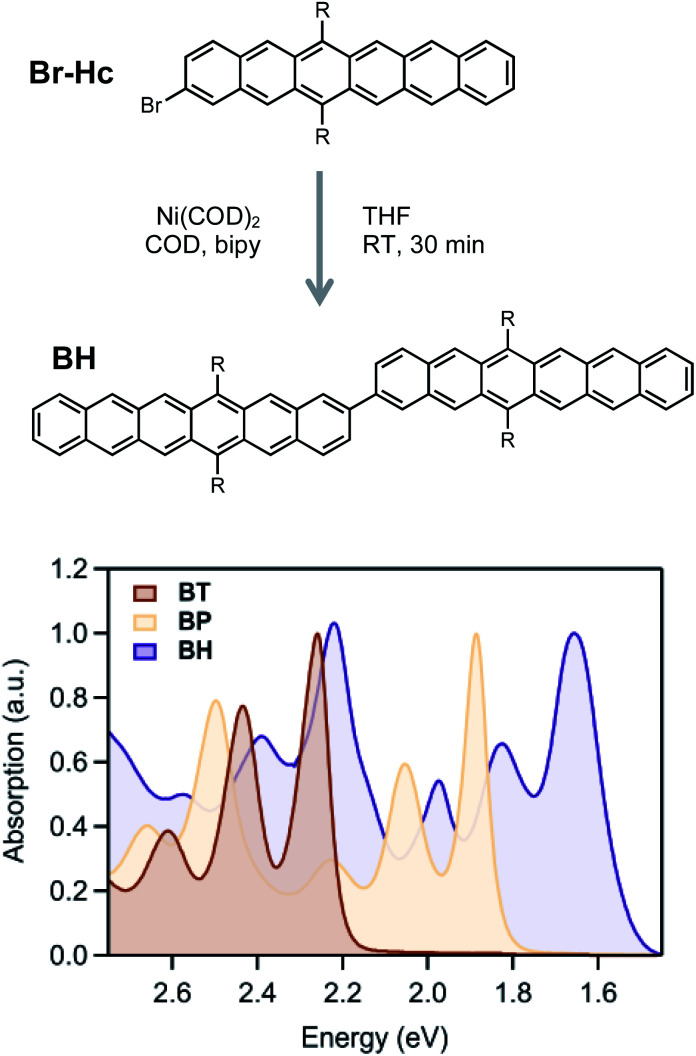
(Top) Key synthetic step to obtain **BH**. (Bottom) Steady-state absorption spectra in the UV-visible region of the hexane dimer **BH** taken in dilute chloroform solution. The spectra of **BP** and **BT** have been added for comparison.

Hexacene derivatives are notoriously unstable and react readily with oxygen or dimerize upon exposure to light. In order to synthesize and characterize these compounds, we adopted the (triisobutyl)silyl acetylene (TIBS) group introduced by Anthony *et al.*, which provides better solubility and stability compared to the more commonly employed triisopropylsilyl acetylene (TIPS) group.^46,57,58^ Additionally, we connected the dimer at the 2-position in order to compare the excited state dynamics with our previously reported **BP**^16^ and **BT**^59,60^ with similar connectivity and protecting groups. While Suzuki–Miyaura cross coupling conditions between brominated and analogous borylated acene partners has been the workhorse strategy for preparing acene dimers, it was crucial to simplify the synthetic manipulation of the relatively unstable hexacene derivatives. Therefore, we adopted a mild and step-economical strategy to perform a homocoupling of 2-bromo (TIBS)hexacene using bis(cyclooctadiene)nickel(0) at room temperature (Fig. 2), forming the hexacene dimer in high yield.

The absorption spectra (Fig. 2) show only a modest bathochromic shift, comparing the monomer, Br-HC, to **BH**, indicating little excited state delocalization over the entire molecule (ESI Fig. S3†), similar to what is observed in **BT** and **BP** derivatives. The energy of the singlet excited state was estimated from the absorption onset to be ∼1.55 eV. Given that the energy of the triplet state in solution phase hexacene is estimated to be ∼0.55 eV,^50,54^ this gives rise to a highly exothermic driving force for fission, approximately double what has been reported for **BP**, the most exothermic previously reported SF dimer.

The singlet fission dynamics in **BH** are established using transient absorption spectroscopy (TAS), following well established techniques.^14,16,18,23,27,52^ These include comparison of directed photoexcited transient species to triplet sensitization experiments. Spectral decomposition using global analysis techniques allows us to readily extract the time constants for singlet fission and the evolution associated spectra consistent with a sequential decay (S_0_ → S_1_ → TT → S_0_). We note that TAS experiments were carried out in anaerobic conditions, deoxygenating the solution by sparging with argon for 5 min, to prevent photooxidative decomposition. The data was collected after excitation (680 nm pump), using ∼100 fs pulses with a fluence of ∼25 μJ cm^−2^. The stability of the compound during the experiment was confirmed by linear absorption measurements and by the repeatability of the measurement over multiple transient absorption data sets.

**Fig. 3:**
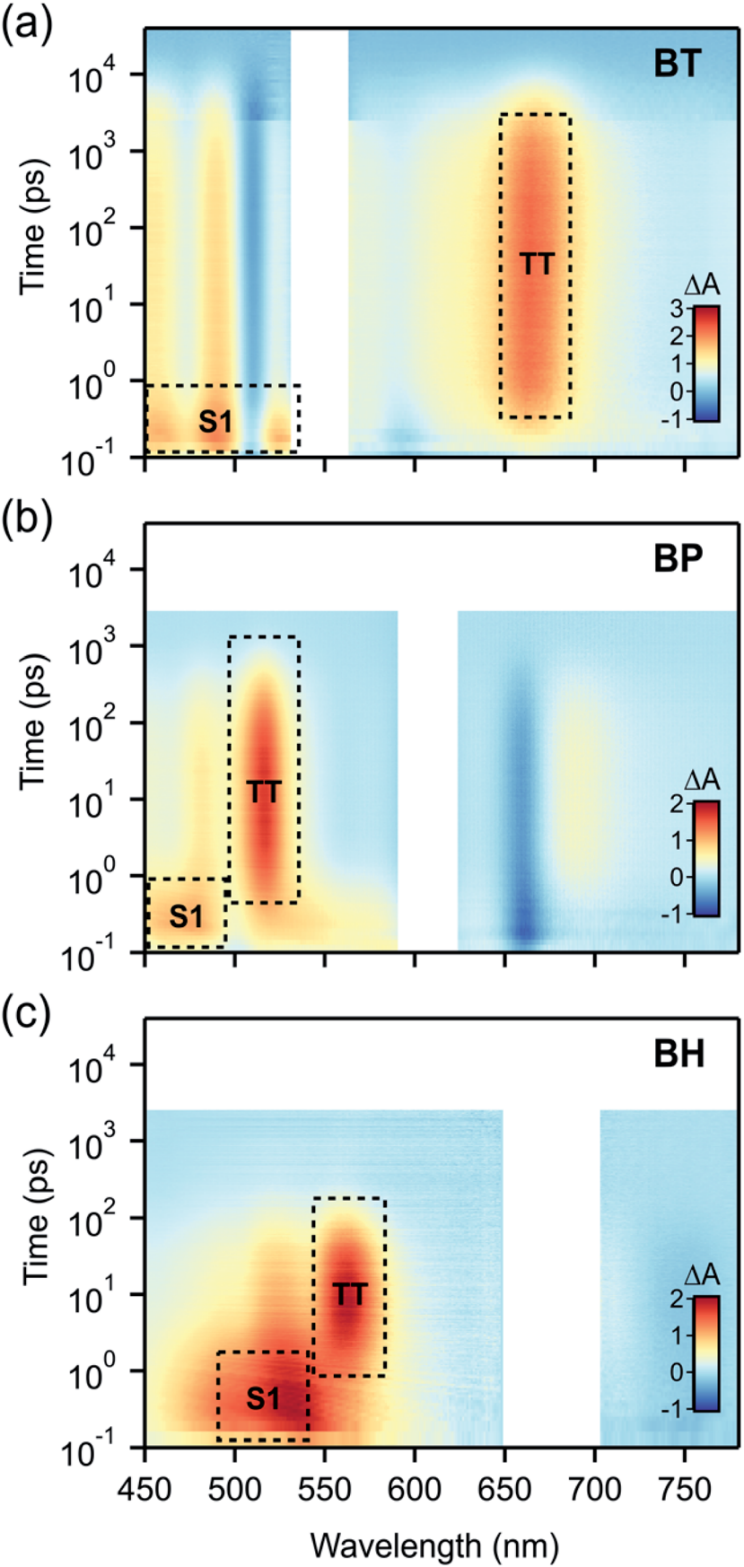
Transient absorption spectra in chloroform for (a) **BT**, (b) **BP** and (c) **BH** excited at 545, 600 and 680 nm respectively. Prominent features of singlet and triplet pair photoinduced absorption have been annotated. In all cases, the triplet pair dynamics are qualitatively similar but show differences in the rate constants for formation and decay.

We find that **BH** undergoes rapid singlet fission to a bound triplet pair, followed by rapid concerted decay of the triplet pair back to the ground state. While these dynamics are qualitatively similar to **BP** and **BT** (Fig. 3), differences in the rate constants elucidate the role of molecular vibrations in both the formation and decay of the biexciton. Photoexcitation of **BH** generates the singlet exciton, which is most readily identified by a characteristic photoinduced absorption (PIA) with a *λ*_max_ near 530 nm. The decay of this state occurs with a time constant of 2.5 ps and is concurrent with the rise of the triplet pair state, which has a characteristic PIA near 560 nm (annotated in Fig. 3).

To verify that triplet pairs are formed *via* singlet fission, spectral decomposition of transient absorption data are compared to triplet-sensitization experiments on **BH**. For triplet sensitization, neat solvent is replaced by a 20 mM solution of anthracene, which upon photoexcitation at 360 nm rapidly generates triplets *via* intersystem crossing. Due its high concentration, essentially all absorption occurs in the anthracene, followed by collisional transfer of triplet excitons to **BH** (data in ESI†). The singlet fission triplet pair spectra (from spectral decomposition) and sensitized spectra are indistinguishable, validating our assignment of the triplet state (ESI Fig. S2†). However, we find that the product of singlet decay is triplet pairs that do not dephase into free triplets in these compounds. The triplet pair can be differentiated from an individual triplet because, despite its spectral similarity, it decays remarkably fast, with a recombination time constant of 104 ps. In contrast, the intrinsic lifetime of a lone triplet state on **BH** was estimated to be 11 μs. These data reflect the dominance of the ^1^(TT) → S_0_ concerted decay process that has been observed in other contiguous dimer systems, and indicates a system in the strong exchange coupling limit.^29^

**Fig. 4:**
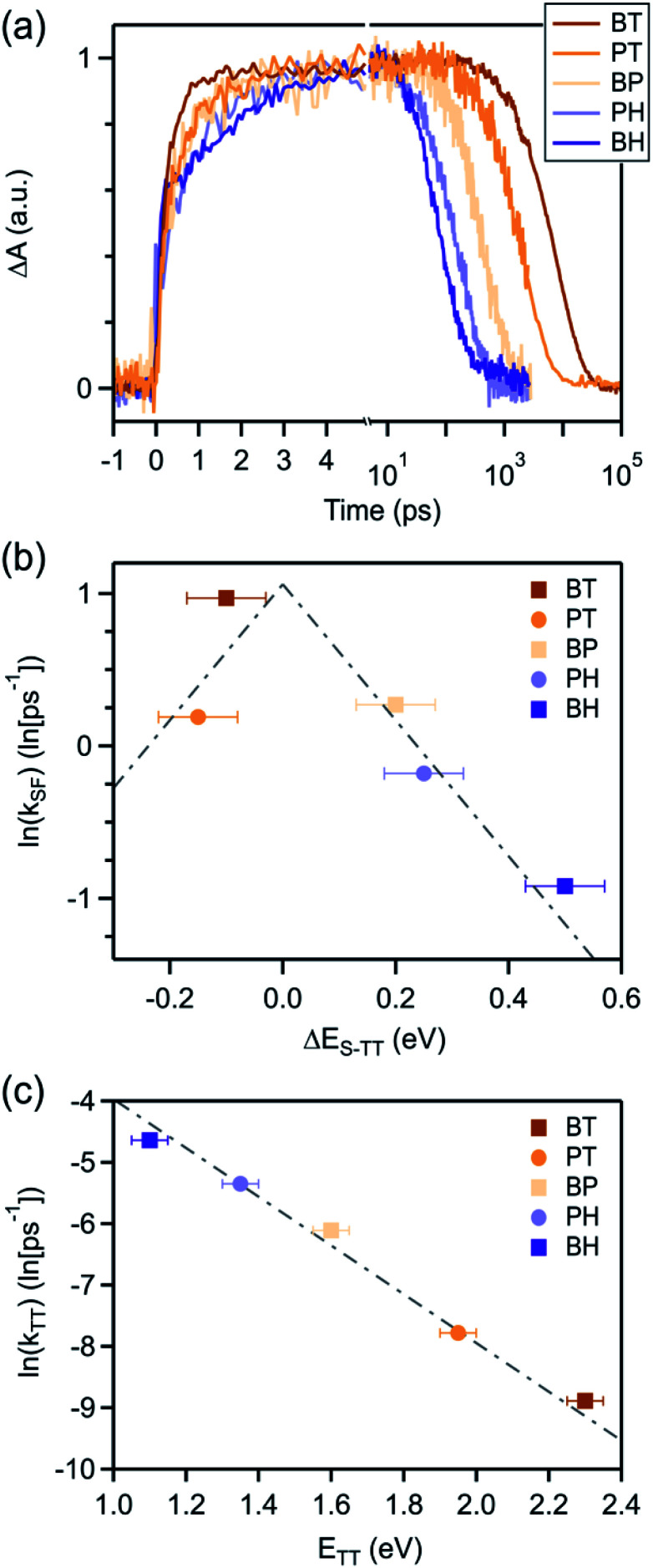
(a) Kinetics at wavelengths selective for triplet photoinduced absorption reveals the slowest triplet rise and fastest triplet decay in **BH**, intermediate rates of rises and decay in **BP**, **PT**, and **PH**, and the fastest triplet rise and slowest triplet decay in the tetracene dimer (**BT**). The natural log of the rate constant for (b) triplet pair formation, *k*_SF_ and (c) triplet pair decay, *k*_TT_ scale approximately linearly (gray lines) with energy offset, consistent with an energy gap law.

We can compare the overall singlet fission dynamics in **BH**, to a set of analogous contiguous dimers with identical connectivity – **BP**, **BT**, **PT**, and **PH** (Fig. 4, summary Table in ESI†). The 2.5 ps time constant for singlet fission in **BH** is considerably slower than the time constants observed in all other contiguous dimers: **BP** (0.76 ps), **BT** (0.38 ps), **PT** (0.83 ps), and **PH** (1.2 ps). This slower time constant in **BH** is indeed consistent with a multi-vibrational dissipation process for the excess energy driving singlet fission, similar to the role of optical phonons in crystalline solids. Unlike analogous molecular crystals, we do not observe instantaneous triplet pair formation *via* a coherent process.^41^ Still, the dependence of the singlet fission rate on the exoergicity implies that vibrations play a large role in the determining the dynamics of the incoherent generation process. In hexacene, electron-phonon coupling proceeds through a few dominant vibrational modes, with energies of ∼180–200 meV.^36,41^ This can be clearly seen in the vibronic overtones in the linear optical absorption spectrum (Fig. 2), with an energy spacing approximately matching this energy. For compounds with singlet–triplet pair energy gaps exceeding 400 meV (as is expected in hexacene), this corresponds to several molecular vibrations and results in a slower overall singlet fission process. The dependence of the singlet fission rate constant (*k*_SF_) on the driving force is not monotonic and peaks for **BT** (Fig. 4b), which has the smallest difference between the singlet and triplet pair. We note that **PT**, which is slightly more endothermic, has a slower time constant. The trend from **BT** > **BP** > **BH** is similar to what is observed in molecular crystals.^36^ While uncertainty in the absolute triplet energies^53,54^ (error bars in Fig. 4b and c) precludes a deeper analysis, we find that a simplified rate scaling adopted from Busby *et al.*^36^ – *k*_SF_ ∼ exp(−Δ*E*_S-TT_) – satisfactorily reproduces the observed trend (dotted lines, Fig. 4b).

Similarly, the triplet pair recombination process appears to depend only on the total energy of the triplet pair, and follows the same simple *k*_TT_ ∼ exp(−Δ*E*_TT-S_0__) scaling behavior (Fig. 4c) observed for *k*_SF_. Here, the wider energy range allows us to confirm that the behavior is dictated by a simple energy gap law, which has previously been observed for other radiationless transition processes, including free triplets.^55,56,61^ The manifestation of the energy gap law here again indicates the importance of molecular vibrations in the overall singlet fission process. We can use evidence from our previous work on molecular dimers to help explain this phenomenon. Contiguous dimers have shown that the singlet and triplet pair energy manifolds are mixed, with allowed TT-S_*n*_ optical transitions.^32,62^ Furthermore, radiationless decay of triplet pairs has been shown to slow down as chromophore proximity decreases. From this, we suggest that the triplet pair decays through coupling to singlet vibronic modes, permitting a rapid and spin-allowed route back to the ground state. We note that the triplet pair lifetime is less than the photoluminescence lifetime of the monomers in all cases, despite the similar energy of the triplet pair to the singlet for **BT** and **BP**.^16,60,63,64^ This supports our assertion of a very distinct recombination process for the triplet pair.

## Conclusions

In conclusion, we report a 2,2′-hexacene dimer **BH**, synthesized through a mild Ni-mediated homocoupling procedure. This compound exhibits sufficient stability for structural and spectroscopic characterization. Using ultrafast transient absorption spectroscopy, we discover relatively slow rates of singlet fission which we attribute to the excessive exothermicity of the hexacene dimer. The resulting triplet pair state is short-lived, explained by the very low ∼1.0 eV energy of the bound state. Our results add clarity to the energy gap law and sheds light on the importance of molecular design as a tool for creating materials with tailored rates of singlet fission and triplet pair recombination.

## Conflicts of interest

There are no conflicts to declare.

## Supplementary Material

SC-011-C9SC05066C-s001
