# Supplementary material for: Singlet fission in a hexacene dimer: energetics dictate dynamics
Source: Chem Sci. 2019 Dec 9;11(4):1079–84. doi: 10.1039/c9sc05066c (PMC8146228; doi:10.1039/c9sc05066c)
Supplement: SC-011-C9SC05066C-s001 [file SC-011-C9SC05066C-s001.pdf]

Electronic Supplementary Material (ESI) for Chemical Science.
This journal is © The Royal Society of Chemistry 2019

Singlet Fission in a Hexacene Dimer: Energetics Dictate Dynamics

Supporting Information

Samuel N. Sanders,[†a] Elango Kumarasamy,[†a] Kealan J. Fallon,[a]

Matthew Y. Sfeir,*[b,c] and Luis M. Campos*[a]

[a] Department of Chemistry, Columbia University, New York, NY 10027, USA

[b] Photonics Initiative, Advanced Science Research Center, City University of New York, New York, NY 10031

[c] Department of Physics, Graduate Center, City University of New York, New York, NY 10016, USA

METHODS

Transient Absorption Spectroscopy: Transient absorption spectroscopy was performed on a setup previously described in detail by our group.[1] Briefly, a commercial Ti:Sapphire laser system (1kHz repetition rate) along with a commercial optical parametric amplifier (TOPAS-C) was used to generate pump pulses with approximately 100 fs pulse widths. For femtosecond measurements, supercontinuum probe light was generated by focusing the 800 nm fundamental into a sapphire disc. The probe light was split into signal and reference beams, which were detected on a shot-by-shot basis by a fiber-coupled silicon visible diode array. The pump-probe delay was controlled by a mechanical delay stage. For longer delay times (ns - μs), a separate sub-ns supercontinuum laser is used to generate probe pulses that are electronically synchronized to the ultrafast laser (Ultrafast Systems EOS). All transient absorption measurements were performed under low excitation fluence conditions (~ 25 $\mu J/cm^2$) in inert (Ar) atmosphere.

Triplet Sensitization: Triplet sensitization studies are performed using nanosecond transient absorption measurements described above. Similar dilute solutions (~ 50 μM) of iSF compounds are used, with the neat solvent replaced by a 20 mM solution of anthracene sensitizer. Resonant pumping of the anthracene at 360 nm and the large excess of anthracene ensures that nearly all photoexcitation occurs in the anthracene. After undergoing intersystem crossing to create triplet excitons on anthracene, collisional transfer populates an individual triplet state on our singlet fission dimers. The triplet transfer time is determined by measuring the decay of the anthracene triplet-triplet absorption signal near 420 nm along with the simultaneous rise of the hexacene triplet-triplet absorption signal near 560 nm. The triplet lifetime is determined from the decay of the hexacene triplet-triplet absorption.

Global Analysis: All transient absorption data was analyzed using the Wavemetrics Igor Pro software package. Briefly, singular value decomposition was used to reduce the size of the data set and identify the minimum number of species and time constants needed to represent the full data set. Global fitting using a parallel decay model (simple sum of exponentials) assuming first order decay of all species, followed by spectral decomposition (often referred to as decay associated spectra or DAS). Finally, a sequential model ($S_0 \rightarrow S_1 \rightarrow TT \rightarrow S_0$) was constructed from the parallel model using matrix diagonalization approaches, allowing for a new set of spectral decompositions that represent the "excited state spectra" (independent of time) of each step in the sequential model.[2] The sequential spectral decompositions, typically referred to as species associate or evolution associated spectra (EAS) are used to identify the singlet and

triplet states. The latter is equivalent to the long time transient spectra in the raw data, and can be directly compared to triplet sensitization spectra for identification.

Triplet Energy Assignment: Assigning triplet energies in acene materials is notoriously difficult. We have taking the best available values from the literature, using experimental values of T_1 = 0.8 eV for TIPS-Pc from phosphorescence measurements and using a value of $^1(TT)$ = 2.3 eV for tetracene from delayed fluorescence.[3] The value for hexacene of T_1 = 0.55 eV is taken from Ref. [4]

TRIPLET SENSITIZATION RESULTS

Triplet sensitization measurements of BH were performed using the procedure described above. The global analysis was used to spectrally deconvolute the data and assign time constants.[2] A sequential model of $S_1 \rightarrow TT \rightarrow S_0$ was sufficient to reproduce the data.

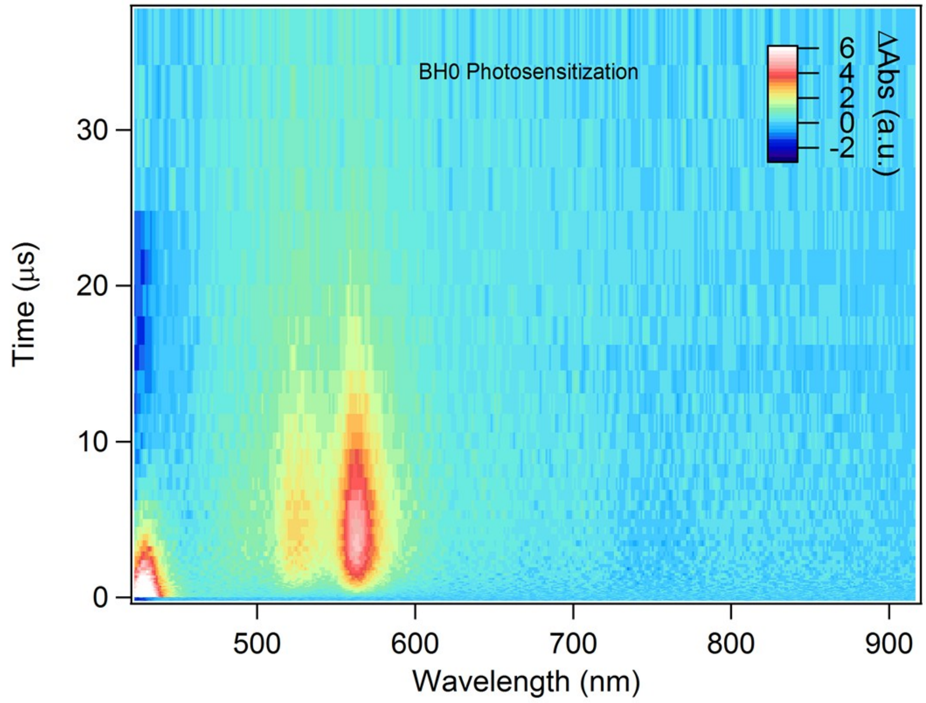

Figure S1. Photosensitization experiments are accomplished by dissolving **BH** along with ~20 mM anthracene in chloroform and preferentially photoexciting the anthracene at 360 nm (~50 μJ/cm^2), which results in intersystem crossing and eventually collisions which populate individual **BH** triplets.

In the photosensitization experiment, an excess of anthracene is dissolved along with the acene dimer. Photoexcitation at 360 nm, followed by intersystem crossing results in a population of anthracene triplets, whose prominent photoinduced absorption can be seen near 420 nm. The decay of this signal occurs concomitant with the rise of the signal near 560 nm, which corresponds to collisional population of the lower energy hexacene triplets on time scales of ~ 1.5 μs. This triplet spectrum is compared to the triplet pair produced by SF in the main text (figure 2), and its lifetime is ~11 μs.

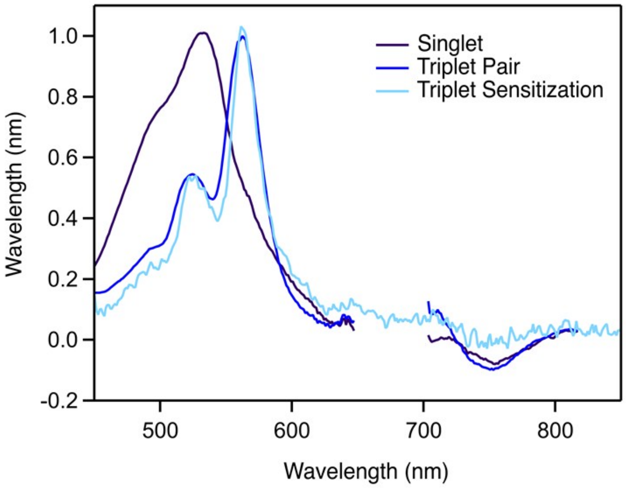

Figure S2. Results of spectral decomposition for direct photoexcitation compared to triplet sensitization experiments.

SUMMARY OF MANUSCRIPT PARAMETERS

Summary of parameters used in this manuscript:

Dimer Components[a]	S_1 (eV)	TT (eV)	$\Delta E_{S\text{-}TT}$	k_{SF} (ps^{-1})	k_{TT} (ps^{-1})
**PT**	1.8	1.95	-0.15	1.20	4.17E-04
**BT**	2.2	2.3	-0.10	2.63	1.37E-04
**BP**	1.8	1.6	0.2	1.32	2.22E-03
**PH**	1.6	1.35	0.25	0.83	4.76E-03
**BH**	1.6	1.1	0.5	0.40	9.62E-03

PT and **PH** are “heterodimers” comprising one unit of Pentacene and Tetracene (**PT**), and one unit of Pentacene and Hexacene (**PH**).

SYNTHESIS

Synthesis scheme of hexacene dimer (**BH**)

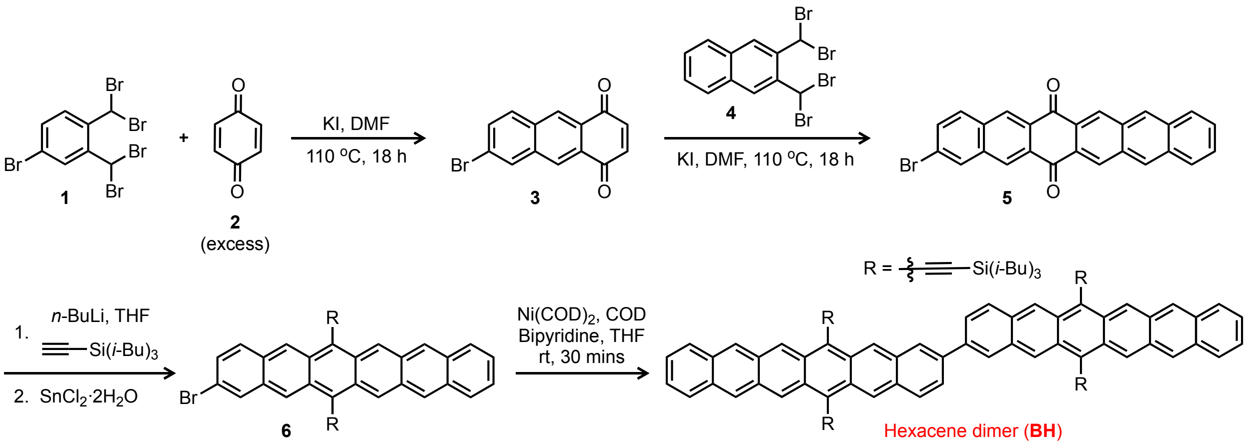

To a solution of $Ni(COD)_2$ (16.1 mg, 0.06 mmol, 1.0 *equiv.*) in THF (2 mL) added COD (12.8 mg, 0.12 mmol, 2.0 *equiv)* and bipyridine (9.2 mg, 0.06 mmol, 1.0 *equiv.*) under N_2 atmosphere during which the solution turned purple. After stirring for 5 mins, added a solution of bromohexacene derivative **6**[5] (50 mg, 0.06, 1.0 *equiv.*) in THF (2 mL). This mixture was stirred at room temperature for 30 mins. The mixture was concentrated under reduced pressure at room temperature and the residue was loaded onto a small alumina column using hexanes. The column was first eluted with hexanes and then with DCM: hexanes mixture (10:90) to obtain a green solid of **BH** in 45 % yield. The chloroform solution of this solid appeared purple in color.

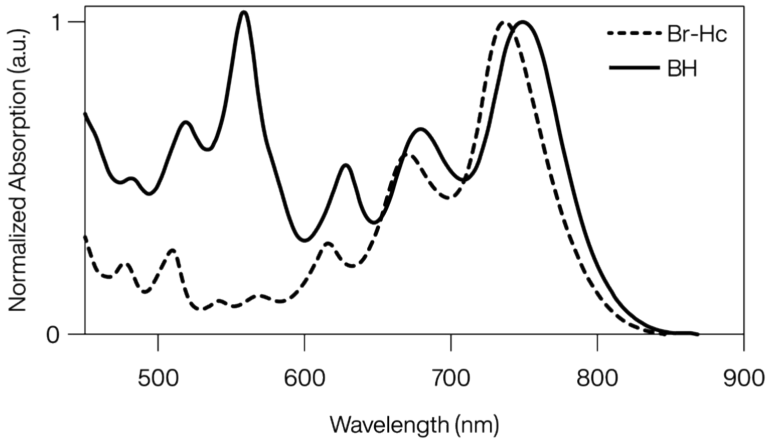

Figure S3. Steady-state absorption spectra in the UV-visible region of the hexacene dimer **BH** (solid line) and the brominated monomer **Br-Hc** (dashed) taken in dilute chloroform.

Note: The yield of the reaction is not optimized. During the reaction, column chromatography and solvent evaporation exposure to light was avoided as much as possible. The solvent evaporation from column chromatography was done below 30 °C. The solid sample of **BH** was stored under argon atmosphere in an aluminum wrapped vial in a freezer.

HRMS (ASAP): Calculated $[M]^+$: 1542.9563; Observed: 1542.9575.

^{1}H-NMR (400 MHz, $CDCl_3$, δ ppm): 9.61 (s, 4H), 9.34 (s, 4H), 9.28 (s, 4H), 8.65 (s, 4H), 8.31 (s, 2H), 8.13-8.10 (m, 2H), 7.99-7.97 (m, 4H), 7.93-7.91 (m, 2H), 7.39-7.37 (m, 4H), 2.35-2.27 (m, 12H), 1.30-1.28 (m, 72H) and 1.09-1.06 (m, 24H).

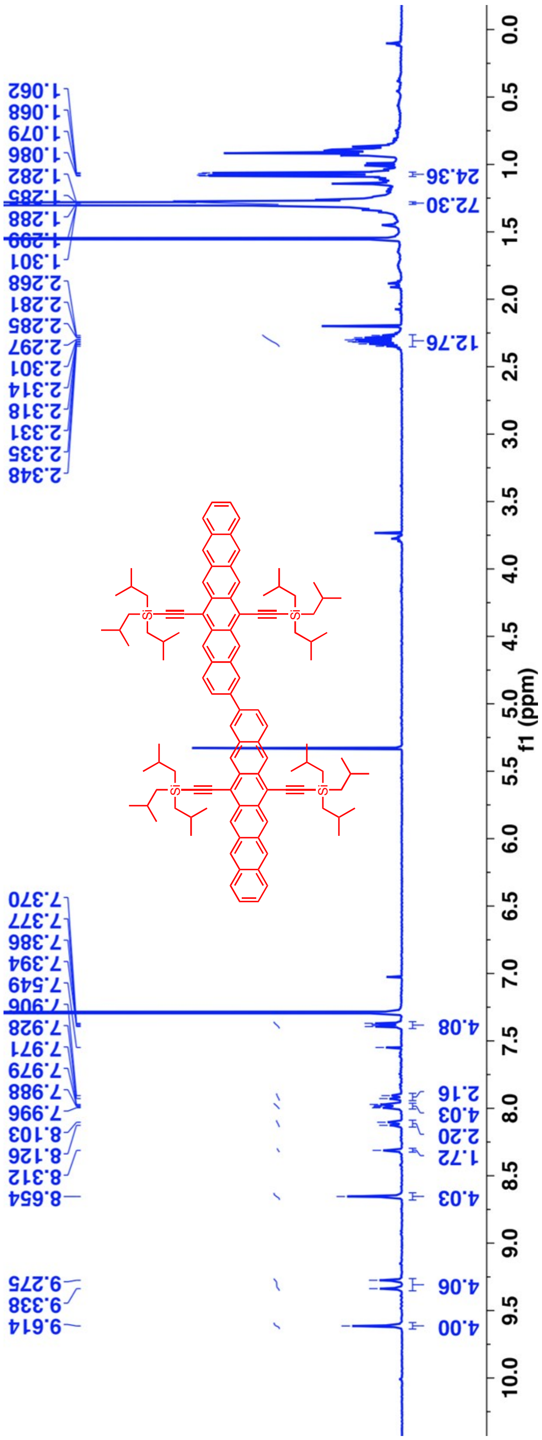

REFERNCES

1. Sanders, S. N.; Kumarasamy, E.; Pun, A. B.; Appavoo, K.; Steigerwald, M. L.; Campos, L. M.; Sfeir, M. Y., Exciton Correlations in Intramolecular Singlet Fission. *J. Am. Chem. Soc.* **2016,** *138* (23), 7289.
2. Snellenburg, J. J. L., S. P.; Seger, R.; Mullen, K. M.; van Stokum, I. H. M., Glotaran: A Java-Based Graphical User Interface for the R Package TIMP. *J. Stat. Soft.* **2012,** *49* (3), 1-22.
3. Yong, C. K.; Musser, A. J.; Bayliss, S. L.; Lukman, S.; Tamura, H.; Bubnova, O.; Hallani, R. K.; Meneau, A.; Resel, R.; Maruyama, M.; Hotta, S.; Herz, L. M.; Beljonne, D.; Anthony, J. E.; Clark, J.; Sirringhaus, H., The entangled triplet pair state in acene and heteroacene materials. *Nature Communications* **2017,** *8* (1), 15953.
4. Angliker, H.; Rommel, E.; Wirz, J., Electronic spectra of hexacene in solution (ground state. Triplet state. Dication and dianion). *Chemical Physics Letters* **1982,** *87* (2), 208-212.
5. Sanders, S. N.; Kumarasamy, E.; Pun, A. B.; Steigerwald, M. L.; Sfeir, M. Y.; Campos, L. M., Intramolecular Singlet Fission in Oligoacene Heterodimers *Angew. Chem. Int. Ed.* **2016,** *55* (10), 3373–3377.
